# Balancing mesh-related complications and benefits in primary ventral and incisional hernia surgery. A meta-analysis and trial sequential analysis

**DOI:** 10.1371/journal.pone.0197813

**Published:** 2018-06-06

**Authors:** Manuel López-Cano, Lidia A. Martin-Dominguez, José Antonio Pereira, Manuel Armengol-Carrasco, Josep M. García-Alamino

**Affiliations:** 1 Abdominal Wall Surgery Unit, Department of General Surgery, Hospital Universitari Vall d’Hebron, Universitat Autònoma de Barcelona, Barcelona, Spain; 2 Department of Surgery, Parc de Salut Mar, Experimental and Health Science, Universitat Pompeu Fabra, Barcelona, Spain; 3 DPhil Programme in Evidence-Based Healthcare, University of Oxford, Oxford, United Kingdom; Cardiff University, UNITED KINGDOM

## Abstract

**Background:**

Primary ventral hernia (PVH) and incisional hernia (IH) repair using a mesh appears to reduce hernia recurrence. However, are the benefits of mesh offset in part by mesh-related complications? The aim of this study was to compare placement of a mesh versus simple suture for recurrence and postoperative complications in the repair of PVH or IH.

**Methods:**

Five databases were searched for randomized controlled trials (RCTs). The study population was patients with a PVH or IH undergoing hernia repair. Intervention was placement of a nonabsorbable synthetic mesh, regardless of mesh location, surgical technique, hernia characteristics or surgical setting compared to primary suture. Primary outcome was the incidence of hernia recurrence. Secondary outcomes were wound infection, hematoma, seroma, postsurgical pain, duration of operation, and quality of life. A random-effects meta-analysis with trial sequential analysis (TSA) was used.

**Results:**

10 RCTs with a total of 1270 patients were included. A significant reduction of the incidence of PVH or IH recurrence using a mesh for repair (risk ratio [RR] 0.39, 95% CI 0.27–0.55; *P* < 0.00001; *I*^2^ = 20%) was observed. TSA for recurrence, the accrued information size (1270) was 312% of the estimated required information size (RIS). Subgroup analysis for PVH and IH confirms reduction of recurrence after using a mesh in both groups. Overall postoperative complications did not show statistically significant differences between the mesh and surgical suture groups (RR 1.31, 95% CI 0.94–1.84; *P* = 0.12; I^2^ = 27%) but the accrued information size was only 22.4% of RIS and by subgroups complications were only related with IH repair.

**Conclusions:**

Evidence for the efficacy of repair of PVH or IH using a nonabsorbable synthetic mesh in terms of recurrence was found to be robust. Evidence for complications remains inconclusive.

## Introduction

Incisional hernias (IH) are mostly repaired by placement of a prosthetic synthetic mesh as compared to simple suturing[1]. Repair methods for primary ventral hernias (PVH) are variable (i.e. mesh or suture) [2]. Data of European registries show that 80–90% of materials used for hernia repair are nonabsorbable synthetic meshes[2]. Other national registries of incisional hernia repair provide similar data and 95% of meshes used for this purpose are of the same nature[3].

Decisions of using a nonabsorbable synthetic mesh versus simple suture can be based on personal experience, analysis of results from observational studies (i.e. data registries), and randomized trials (i.e. randomized controlled trials [RCTs]). The analysis of registries with long-term follow-up data shows that the use of a mesh as compared with primary suture appears to reduce hernia recurrence, but the number of complications (seroma, infection, etc.) may be increased[4]. Recent results of systematic reviews and meta-analyses of RCTs concluded that the use of a nonabsorbable synthetic mesh for the surgical repair of PVH or IH reduces the number of recurrences[5,6]. These benefits, however, are also associated with a higher incidence of complications, such as seroma or surgical site infection [5] and seem to be associated with a risk of chronic postsurgical pain[6].

Although data provided by registries and meta-analyses are necessary for decision-making, the interpretation and generalizability of results may be difficult. Registries are limited by the observational nature of data[4], and meta-analysis although placed at the top of the evidence pyramid[7], may not be statistically reliable because of false positive results (type I error) or overestimation of the effect of treatment due to systematic errors (bias) or random errors related to repeated measurements[8]. Trial sequential analysis (TSA) controls the risk for type I errors and helps to clarify whether additional trials are needed[9]. However, TSA methodology is not frequently used in the design of meta-analyses and some authors recommend the systematic use of TSA to determine whether a firm and solid evidence can be obtained[8,9].

The objective of the present study was to perform a meta-analysis with TSA of randomized clinical trials to assess the efficacy and postoperative complications of placement of a nonabsorbable synthetic mesh versus simple suture in the surgical repair of abdominal wall defects (i.e. PVH or IH).

## Methods

The study protocol for this meta-analysis and TSA was conceived, approved and developed by members of the Department of General Surgery of Hospital Universitari Vall d’Hebron (Barcelona, Spain). This meta-analysis was carried out according to the predefined methodological criteria outlined in the Preferred Reporting Items for Systematic Reviews and Meta-Analysis (PRISMA) statement[10].

### Systematic literature search

We conducted a systematic literature search of MEDLINE (PubMed), SCOPUS, CINAHL, WOK (Web of Knowledge), and Google Scholar. Search strategy was based on combinations of Medical Subject Heading (MeSH) terms and text words for each database, as shown in Box 1 for Medline. The search was limited to randomized controlled trials (RCTs). No publication date restrictions before November 2016 were applied. We included studies published in English, French, German, and Spanish languages. The reference lists of all retrieved studies were cross-checked for additional reports. The literature search was performed independently by two authors (M L-C and LA M-D). Any discrepancies between the two reviewers were discussed with a third reviewer (J G-A) and solved by consensus.

**Box 1. Search strategy for the Medline database**

((((((((""Hernia, Incisional"" OR ""Hernias, Incisional"" OR ""Incisional Hernias"" OR ""Postoperative Hernia"" OR ""Hernia, Postoperative"" OR ""Hernias, Postoperative"" OR ""Postoperative Hernias"") AND ((Meta-Analysis[ptyp] OR Randomized Controlled Trial[ptyp] OR Review[ptyp] OR systematic[sb]) AND (English[lang] OR Spanish[lang] OR French[lang] OR German[lang])))) OR ((""Hernias, Ventral"" OR ""Ventral Hernias"" OR ""Ventral Hernia"") AND ((Meta-Analysis[ptyp] OR Randomized Controlled Trial[ptyp] OR Review[ptyp] OR systematic[sb]) AND (English[lang] OR Spanish[lang] OR French[lang] OR German[lang])))) AND ((Meta-Analysis[ptyp] OR Randomized Controlled Trial[ptyp] OR Review[ptyp] OR systematic[sb]) AND (English[lang] OR Spanish[lang] OR French[lang] OR German[lang])))) AND ((((""mesh"") OR ""surgical meshes"") OR ""surgical mesh"") AND ((Meta-Analysis[ptyp] OR Randomized Controlled Trial[ptyp] OR Review[ptyp] OR systematic[sb]) AND (English[lang] OR Spanish[lang] OR French[lang] OR German[lang])))) AND ((Meta-Analysis[ptyp] OR Randomized Controlled Trial[ptyp] OR Review[ptyp] OR systematic[sb]) AND (English[lang] OR Spanish[lang] OR French[lang] OR German[lang])))"

### Study selection and data extraction

We established the inclusion criteria for study selection according to the PICOS approach. The population consisted of patients with a PVH or IH undergoing hernia repair. The intervention consisted of placement of a nonabsorbable synthetic mesh, regardless of mesh location, surgical technique, hernia characteristics or surgical setting compared to primary suture of the PVH or IH without mesh.

Primary outcome was the incidence of hernia recurrence diagnosed on clinical grounds by physical exam or radiological evaluation (computed tomography [CT] scan or ultrasonography). Secondary outcomes were short run complications as wound infection, hematoma and seroma. Postsurgical pain, length of operation for each type of surgical procedure, and health-related quality of life.

Two authors (M L-C and LA M-D) independently extracted data from the included trials. Any divergences during the data extraction phase were resolved through discussion with a third investigator (JM G-A).

An *ad hoc* data collection form with the items evaluated was used. For duplicate data reported by the same author(s), the study with the longest follow-up was selected. The randomization method and blind assessment of results according the criteria of Cochrane Collaboration’s tool [11] were used to assess the risk of bias (quality) of included studies in the review. If randomization method and blind assessment were not stated, the study was considerer of low quality.

### Statistical analysis

A meta-analysis pooling the results from PVH and IH for the primary outcome was performed. Additionally, pooled postoperative complications were meta-analyzed in separate and also grouped to estimate the overall effect of complication events.

Sensitive analysis within subgroups (i.e. PVH and IH) for recurrence and postoperative complications (separate and grouped) was planned. Meta-analysis that combine other subgroups (mesh location, hernia characteristics or surgical setting) and meta-analysis combining high quality studies only were also planned.

A random-effects model was used and because data were sparse (number of events and study size) a Mantel-Haenszel statistical method was selected[12]. Groups with zero events were adjusted with a constant continuity adjustment of 0.5 in each arm (as per default adjustment in the RevMan 5.3 software)[13]. All outcomes were expressed as risk ratios (RRs) with 95% confidence interval (CIs). Heterogeneity in the included studies was evaluated using *I*^2^ statistic, with *I*^2^ values of 25%, 25–50%, and > 50% corresponding to small, moderate, and high heterogeneity, respectively[14]. *P* values were also calculated. The Review Manager 5.3 software (Copenhagen: The Nordic Cochrane Centre, The Cochrane Collaboration, 2013) was used for analysis.

Sequential multiplicity (repeated updates) and sparse data increased the risk of type I error and to control this we performed a TSA[9]. TSA combines an estimate of required information size (RIS) for meta-analysis with monitoring boundaries used as thresholds for statistical significance. The less data accumulated, the more conservative was the TSA boundaries, making it less likely to declare statistical significance before the RIS has been reached. Similar to a sample size calculation for a single trial, estimating RIS involves a calculation that includes type I error, type II error, the control event rate (CER-baseline risk), and the effect size (relative risk reduction- RRR). The calculation of RIS also requires an estimate of heterogeneity. For the present analysis, we estimated the RIS using the following assumptions: 0.05 for type I error and 0.20 for type II error. The CER was calculated in all meta-analyses as the median of the proportion of events in the control group (non-mesh group). The effect size (RRR) estimated from the included studies was used to estimate the RIS. We used the *I*^2^ present in the included trials as the estimate for heterogeneity. The TSA can be interpreted by viewing the boundaries and whether the cumulative meta-analysis has crossed them. TSA was performed using the TSA software v0.9 (www.uct.dk/tsa/index.html).

## Results

**Fig 1:**
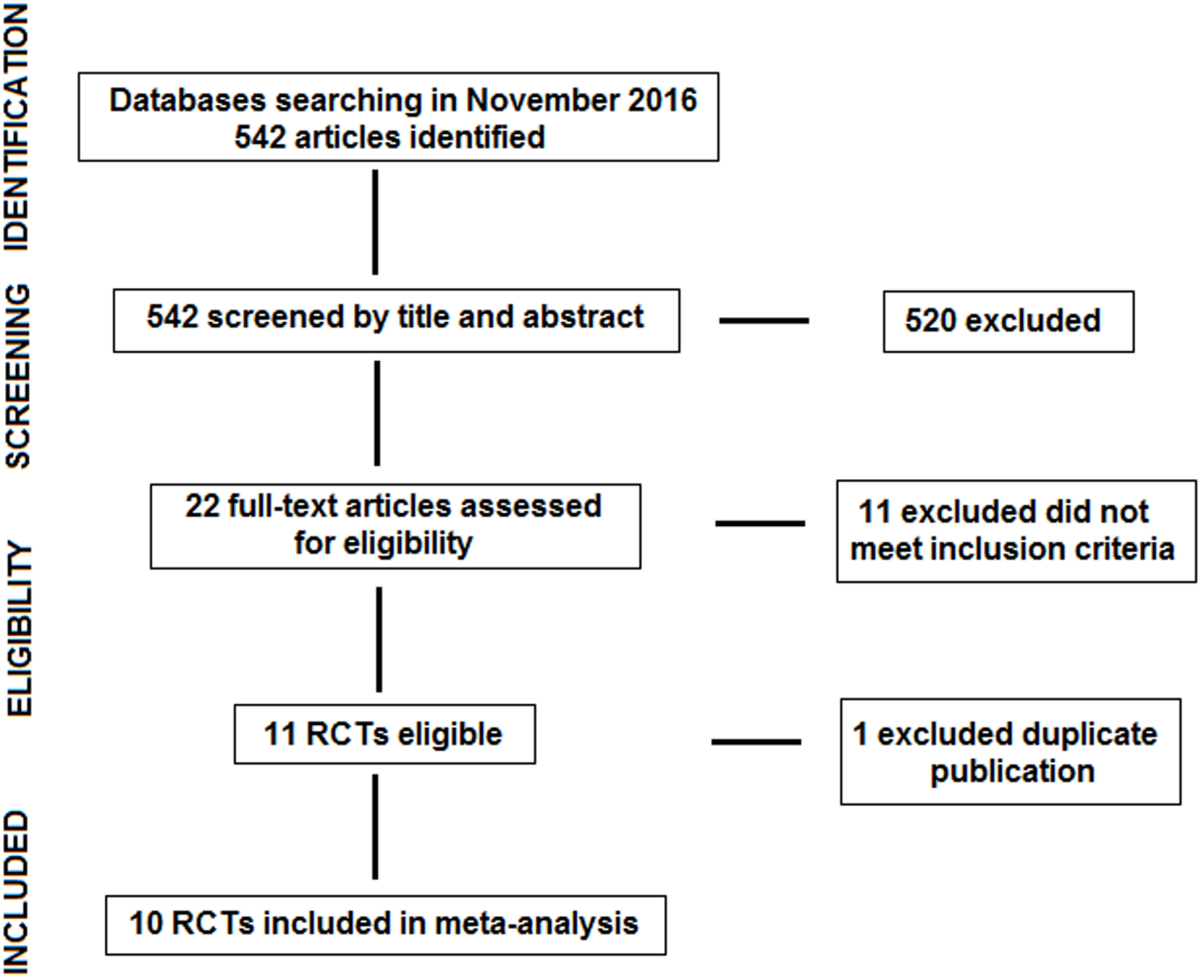
PRISMA flow diagram.

**Fig 2:**
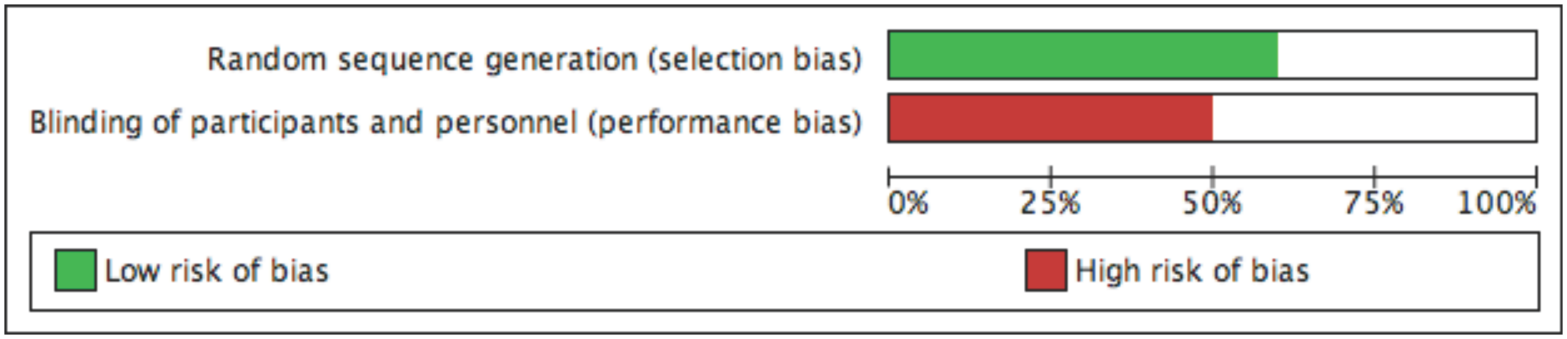
Risk of bias assessment as percentages across all included studies.

Database searching identified 542 articles, 520 of which were excluded after screened by title and abstract. The resulting 22 articles were read in full and assessed for eligibility excluding 11 articles because they did not meet the inclusion criteria. Of the remaining 11 articles[15–25], the study of Luijendijk et al.[16] was excluded because of duplicate data of the same authors, and the article with the longest follow-up was selected[19]. Finally, we included 10 RCTs with a total of 1270 patients[15,17–25] (Fig 1). The characteristics of the included studies, type of hernia, surgical technique (hernioplasty, herniorrhaphy), follow-up, and use of antibiotic prophylaxis are shown in Table 1. After risk of bias assessment six studies were considerer of high quality [17,18,22–25] and four of low quality [15,19–21]. Risk of bias assessments as percentages across all included studies are also presented in Fig 2.

**Table 1:** Characteristics of the 10 RCTs included in the meta-analysis.

Author, year	Characteristics of the trials								
	Randomization	Blinding	No. patients	Type of hernia/surgical setting	Hernia size suture/ mesh	Hernioplasty type/position of mesh	Herniorrhaphy type of suture	Follow-up months	Antibiotic prophylaxis
Arroyo et al[17], 2001	Computerized random number list	No	200	PVH /elective	>3cm (30/32) <3cm (70/68)	Polypropylene/extraperitoneal	Nonabsorbable	64	Yes
Korenkov et al[18], 2002	Sealed envelopes with consecutive numbers	No	103	IH/elective	3.6**±**1.7)/11.7**±**9.4cm	Polypropylene/onlay	Nonabsorbable	9	Yes
Burger et al[19], 2004	Not stated	Not stated	181	IH/elective	20cm^2^ /24cm^2^	Polypropylene/sublay	Nonabsorbable	75–81	Yes
Polat et al[20], 2005	Not stated	Not stated	50	PVH/elective	?	Polypropylene/onlay-sublay	Nonabsorbable	22	Yes
De Vries et al[21], 2007	Not stated	Not stated	35	IH/elective	15/17cm	Polytetrafluoroethylene/Intraperitoneal	Absorbable	36	Yes
Abdel-Baki et al[22], 2007	Sealed envelopes	No	42	PVH/emergency	4.5/4.7cm	Polypropylene/onlay	Nonabsorbable	16	Yes
Ammar et al[23], 2010	Computerized random number list	No	72	PVH/emergency	5.7/5.4cm	Polypropylene/onlay	Nonabsorbable	6	Yes
Venclauskas et al[15], 2010	Not stated	Not stated	161	IH/elective	88.7cm^2^/114.5cm^2^	Polypropylene/onlay-sublay	Nonabsorbable	12	Yes
Weber et al[24], 2010	Computerized random number list	Not stated	364	IH/elective	<25cm^2^/>25cm^2^	Polypropylene/onlay-sublay	Nonabsorbable	> 12	Yes
Lal et al[25], 2012	Computerized random number list	No	62	PVH/elective	?	Polypropylene/sublay	Nonabsorbable	12	Yes

PVH: Primary ventral hernia. IH: Incisional hernia

**Fig 3:**
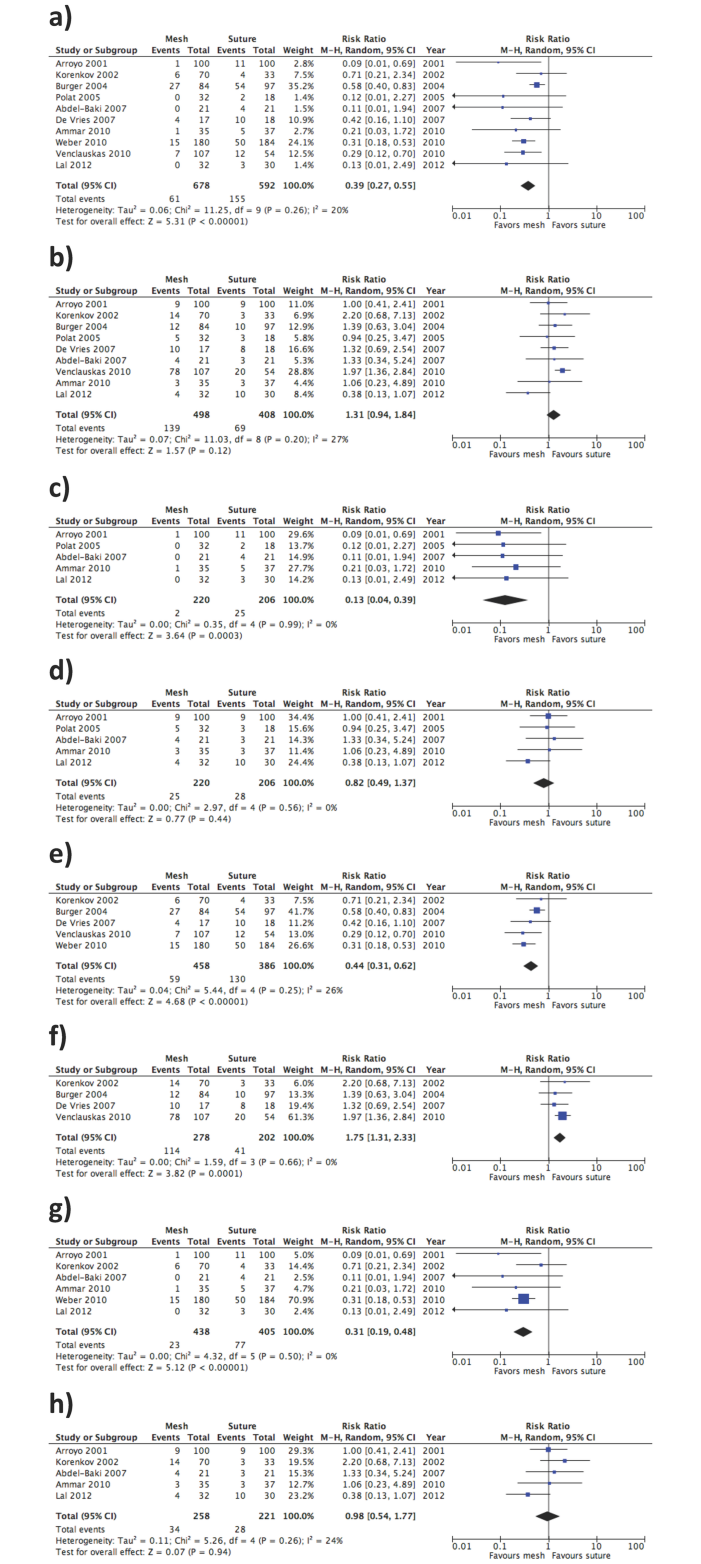
Effect of placement of a mesh for a) prevention of the incidence of PVH and IH recurrence; b) PVH and IH incidence of grouped complication events (wound infection, hematoma, and seroma); c) prevention of the incidence of PVH recurrence; d) PVH incidence of grouped complication events (wound infection, hematoma, and seroma); e) prevention of the incidence of IH recurrence; f) IH incidence of grouped complication events (wound infection, hematoma, and seroma) g) prevention of the incidence of PVH and IH recurrence for high quality studies only; h) PVH and IH incidence of grouped complication events (wound infection, hematoma, and seroma) for high quality studies only.

The meta-analysis for the primary outcome (incidence of PVH and IH recurrence) included all 10 RCTs[15,17–25] and showed a significant reduction of the incidence of hernia recurrence using a prosthetic mesh for hernia repair (RR 0.39, 95% CI 0.27–0.55; *P* < 0.00001; *I*^2^ = 20%) (Fig 3).

The results of meta-analysis for secondary outcomes were as follows: for wound infection reported in 9 trials[15,17–23,25], no statistically significant differences between groups (mesh *vs*. primary suture) were found (RR 1.26, 95% CI 0.83–1.91; *P* = 0.28; *I*^2^ = 0). For postoperative hematoma, the meta-analysis of 7 trials[15,17–20,21,25] showed no statistically significant differences between groups (RR 0.58, IC 95% 0.24–1.37; *P* = 0.21; *I*^2^ = 21%). For development of a seroma, the meta-analysis of 7 trials[15,17,18,20–22,25] showed a significant reduction of the incidence of seroma using sutures (RR 2.44, 95% CI 1.43–4.14; *P* < 0.001; *I*^2^ = 0). The meta-analysis of overall postoperative events grouped as wound infection, hematoma, and seroma including 9 trials[15,17–23,25], did not show statistically significant differences between the mesh and surgical suture groups (RR 1.31, 95% CI 0.94–1.84; *P* = 0.12; I^2^ = 27%) (Fig 3).

Subgroup analysis for recurrence in PVH included 5 RCTs[17,20,22,23,25] and showed a significant reduction of the incidence of hernia recurrence using a prosthetic mesh for hernia repair (RR 0.13, 95% CI 0.04–0.39; *P* = 0.0003; *I*^2^ = 0%) (Fig 3). The results for secondary outcomes in PVH subgroup showed no statistical differences in complications, both separately (Infection: RR 0.69 (0.33–1.49), *P* = 0.34; *I*^2^ = 0%, 5 studies included[17,20,22,23,25]; Hematoma: RR 0.42 (0.11–1.64), *P* = 0.21; *I*^2^ = 0%, 3 studies included[17,20,25]; Seroma: RR 1.66 (0.63–4.36); *P* = 0.30; *I*^2^ = 0%, 4 studies included[17,20,22,25]) or combined (RR 0.82 (0.49–1.37), *P* = 0.44; *I*^2^ = 0%, 5 studies included[17,20,22,23,25]) (Fig 3).

Subgroup analysis for recurrence in IH included 5 RCTs [15,18,19,21,24] and showed a significant reduction of the incidence of hernia recurrence using a prosthetic mesh for hernia repair (RR 0.44, 95% CI 0.31–0.62; *P* < 0.00001; *I*^2^ = 26%) (Fig 3). Analysis of complications in the IH subgroup showed no differences between groups regarding infection (RR 1.62, 95% CI 0.99–2.67; *P* = 0.06; *I*^2^ = 0%, 4 studies included[15,18,19,21]) and hematoma (RR 0.58, 95% CI 0.16–2.11; *P* = 0.41; *I*^2^ = 38%, 4 studies included[15,18,19,21]). However, seroma was statistically significant lower in the suture group (RR 2.88, 95% CI 1.52–5.42; *P* < 0.001; *I*^2^ = 0%, 3 studies included[15,18,21]). The meta-analysis of overall postoperative events grouped as wound infection, hematoma, and seroma in IH subgroup did show statistically significant differences of more complications in the group of mesh (RR 1.75, 95% CI 1.31–2.33; *P* = 0.0001; *I*^2^ = 0%, 4 studies [15,18,19,21]) (Fig 3).

Subgroup analysis for the primary outcome (incidence of PVH and IH recurrence) of high quality studies included 6 RCTs [17,18,22–25] showed a significant reduction of the incidence of hernia recurrence using a prosthetic mesh for hernia repair (RR 0.31, 95% CI 0.19–0.48; *P* = 0.00001; *I*^2^ = 0%), with a similar size of the effect that when all studies (i.e. high and low quality) are included. (Fig 3). Subgroup analysis of overall postoperative events grouped as wound infection, hematoma, and seroma including 5 trials of high quality [17,18,22,23,25] showed no statistical differences in combined complications (RR 0.98, 95% CI 0.54–1.77; *P* = 0.94; *I*^2^ = 24%) (Fig 3).

The incidence of postsurgical pain, duration of operation for each type of surgical procedure, quality of life was not analyzed because of the lack of information regarding these variables. Also, mesh location, hernia characteristics or surgical setting was not analyzed because of the lack of information or were too varied to allow for adequate analysis. In addition, other subgroup analysis by specific type of hernia (PVH or IH) using high quality studies only were not done because of the lack of enough information. Because only 10 RCTs were included in the present meta-analysis, publication bias was not evaluated[26].

**Fig 4:**
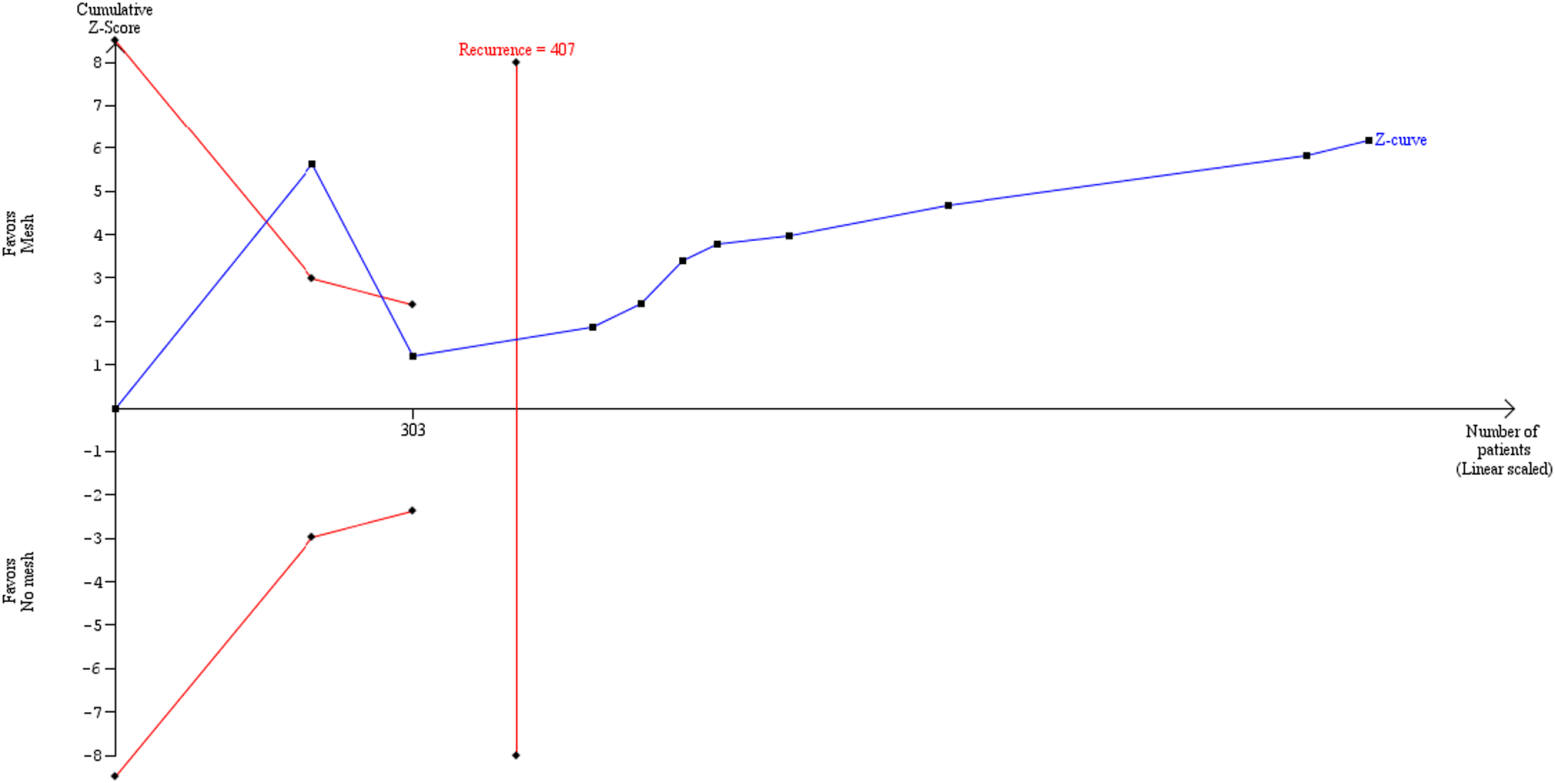
TSA curve for the primary outcome, incidence of recurrence of primary ventral hernia and incisional hernia.

For the primary outcome (incidence of recurrence of PVH and IH), the TSA estimation using a RRR of 61% and a CER proportion of 16% with *I*^2^ of 20%, the accrued information size (1270) was 312% of the estimated RIS (RRR 61% = 407) (Fig 4). For the secondary outcome (PVH and IH complications-infection+hematoma+seroma) the accrued information size (906) was only 22.4% of the estimated RIS (RRR 31% = 4035). The values of the present meta-analysis and TSA for the primary and secondary outcomes and by subgroups including high quality studies analysis are shown in Table 2.

**Table 2:** Results of meta-analysis and values of trial sequential analysis (TSA) for primary and secondary outcomes and by subgroups.

OUTCOME	META-ANALYSIS		TSA VALUES	
	No. patients (AIS)	Risk ratio, 95% CI *I*^*2*^, *P*	Scenario^a^	Estimated RIS (% AIS with respect to the estimated RIS)
**Recurrence**				
PVH + IH	1270	0.39 (0.27–0.55) *I*^2^ = 20%, *P* <0.00001	CER 16%, RRR 61%	407 (312)
PVH	426	0.13 (0.04–0.39) *I*^2^ = 0%, *P* = 0.0003	CER 11%, RRR 87%	200 (213)
IH	844	0.44 (0.31–0.62) *I*^2^ = 26%, *P* <0.00001	CER 27%, RRR 66%	193 (437)
**Complications (Infection + hematoma + seroma)**				
PVH + IH	906	1.31 (0.94–1.84) *I*^2^ = 27%, *P* = 0.12	CER 14%, RRR 31%	4035 (22.4)
PVH	426	0.82 (0.49–1.37) *I*^2^ = 0%, *P* = 0.44	CER 14%, RRR 18%	5497 (7.7)
IH	480	1.75 (1.31–2.33) *I*^2^ = 0%, *P* = 0.0001	CER 23%, RRR 75%	229 (209)
**High quality studies**				
Recurrence (PVH+IH)	843	0.31 (0.19–0.48) I^2^ = 0, *P*<00001	CER 14%, RRR 68%	291 (289.6)
Complications (Infection + hematoma + seroma) (PVH+IH)	479	0.98 (0.54–1.77) I^2^ = 24, *P* = 0.94	CER 9%, RRR 2%	1034802 (0.04)

Abbreviation: PVH, primary ventral hernia; IH, incisional hernia; AIS, accrued information size; RIS, required information size; CER, control event rate; RRR, relative risk reduction.

^a^ Estimated RIS with 0.05 for type I error and 0.20 for type II error; CER in all meta-analysis was the median of the proportion of events in the control group (suture or non-mesh); estimated RRR of the included studies was used for estimating RIS. Suture group is the reference group

## Discussion

Our meta-analysis provides robust evidence in favor of the use of a nonabsorbable synthetic mesh in surgical repair of PVH and IH for the prevention of hernia recurrence. A 61% reduction of the incidence of global recurrence was found. The estimated accrued information size of 407 patients was exceeded by 1270, which was the number of patients included in the meta-analysis. In relation to secondary outcomes and when all complication events were grouped, placement of a mesh for PVH and IH repair did not increase significantly the incidence of wound infection, hematoma, and seroma, although in this case the number of patients analyzed was only 22.4% of RIS. On the other hand, meta-analysis and TSA by subgroups (i.e. PVH and IH) confirms the robust efficacy of a mesh for the prevention of hernia recurrence with 87% and 56% of reduction in hernia recurrence for PVH and IH respectively. Interestingly PVH repair complications (separately or grouped) did not show any difference between mesh and suture although the number of patients analyzed was only 7.7% of RIS. However, IH repair with mesh was related with an increased incidence in complications, grouped or separately (i.e. seroma) with an accrued information size of 209% of RIS.

The framework and recommendations for surgical innovation of a medical device could be based on the IDEAL recommendations (Idea, Development, Exploration, Assessment and Long Term Study)[27,28]. This rational approach for evaluating the use of medical devices includes first, the idea where proof of concept is the key purpose; second, development and exploration of safety; third, assessment of comparative efficacy; and fourth, long-term monitoring of effects. The present meta-analysis would be included in the third stage and demonstrates the efficacy of a mesh in PVH and IH repair to reduce the incidence of recurrence. The evidence is firm because TSA estimation shows that the meta-analysis exceeds RIS [29,30]. However, RCTs included in the meta-analysis reported mid- and short-term results, with long-term results described in only two trials[17,19]. The long-term effect of mesh insertion on hernia recurrence (minimum 5 years of follow-up and corresponding to the fourth stage of IDEAL recommendations) was evaluated in 2003 in a single population-based study of 10822 Washington state patients[31]. This study showed that cumulative recurrence rates increased lineally over the years for incisional hernia repair with or without synthetic material (mesh). Recently, in 2016, registry-based nationwide data of all elective incisional hernia repairs carried out in Denmark from January 1, 2007, to December 31, 2010 were published[4]. A total of 2876 patients with IH repair with mesh were included. Sutured repair was associated with a higher risk of reoperation for recurrence over 5 years compared with mesh repair. Long-term studies may suggest that prosthetic meshes may only delay for years the development of recurrent hernia and its use would only be a palliative treatment of a complex disease[1]. The use of mesh in PVH and IH repair has shown an indisputable positive benefit on daily living of many patients, and because mesh implantation in the mid- and short-term is more effective than suture, as confirmed in our meta-analysis and subgroups analysis, we believe that this surgical approach (i.e. mesh) should continue to be used. It is probable that only a full knowledge and understanding of the biologic process that determines the appearance of a PVH or IH would be the answer for establishing the most appropriate surgical treatment.

Postoperative complications are also a key aspect in the analysis of results of the use of nonabsorbable synthetic meshes. Our meta-analysis of grouped complications (wound infection, hematoma, and seroma) based on all papers included did not show significant differences between mesh and suture groups. Additionally, for this general group the accrued information size of TSA should be at least 4035 patients to obtain reliable conclusions for this outcome and patients of studies included in the meta-analysis accounted for only 22.4% of the information size. After subgroup analysis mesh associated complications were only confirmed for IH, matching data of registers from this type of hernia[4] and for IH the accrued information size of TSA was enough (209% of the information size) suggesting a firm evidence that complications of IH are not interchangeable with PVH complications as a procedure point of view. Postoperative seroma was associated with IH surgery and is a common complication [32] and different factors may contribute to seroma formation, including foreign body reaction to meshes [33]. However, the vast majority of seromas do not exhibit clinical repercussion and are resorbed without sequelae, and only a small percentage of cases require some intervention [34]. Regarding PVH complications did not show any difference between mesh and suture nevertheless the accrued information size of TSA should be at least 5,497 patients (accrued information size only 7.7% of the information size) to obtain reliable conclusions. Accordingly, evidence regarding mesh-related complication in the short-, mid-, and long-term is inconclusive, although only 2 of the 9 RCTs had a long follow-up of 64 and 75–81 months, respectively[17,19].

The analysis regarding only high quality studies confirmed the efficacy of mesh in reducing recurrence of hernia, with a robust evidence, since the accrued information size of TSA was enough (289.6% of the information size), suggesting that low quality studies excluded from this analyses did not overestimated the effect. However, the analysis of high quality studies in relation to complications did not show significant differences between mesh and suture groups and the accrued information size of TSA should be at least 1034802 patients (accrued information size only 0.04% of the information size) to obtain reliable conclusions, confirming that data regarding mesh-related complication remain inconclusive.

There are several limitations in our study. First of all, 4 of 10 RCTs included in the study did not report details of randomization and 5 of 10 did not report blinding, suggesting a low quality of trials and difficulties in excluding a performing and selection bias. Secondly, data of the present meta-analysis are only applicable to patients undergoing PVH or IH repair using a nonabsorbable synthetic mesh, so that results cannot be generalized to other types of prosthetic materials such as biologic or absorbable meshes. Thirdly, only two RCTs included [22,23] were related with emergency condition and contaminated field, so that results are reduced to clean cases and cannot be generalized to emergency and contaminated or clean-contaminated settings because the lack of information. Also, mesh location and hernia characteristics are likely to be confounders but were not analyzed because the data in the studies included did not uniformly reported. Fourth, in the analysis of high quality studies, PVH and IH were clumped with potential bias derived from this limitation. Fifth, because of insufficient information there was no distinction in the analyses between different types of sutures (fast, slow and non-absorbable) and if suture material might affect results. Sixth, because the number of RCTs does not allow adequate assessment of publication bias, it is impossible to determine whether there was an overestimation or underestimation of the beneficial or harmful effect of the intervention due to selective publication of studies. Seventh, other secondary outcomes such as postsurgical pain and quality of life were not analyzed due to the lack of information on these variables. Eighth, mesh related complications were mostly evaluated in short run follow-up and poorly defined and a subset of patient reported outcomes measures (PROMS) was not used.

This meta-analysis has also strengths. Firstly, despite clinical heterogeneity secondary to intrinsic differences of patients and treatments and methodologic heterogeneity (i.e. control of bias), the efficacy of mesh to reduce recurrent hernia was consistent in all studies, as shown by a low statistical heterogeneity (*I*^2^ = 20%). After subgroup analysis this heterogeneity remained lower or non-existent (26% for IH and 0% for PVH). Also in the analysis of complications heterogeneity was lower or non-existent. The statistical homogeneity of studies included in the meta-analysis reinforces the validity of results[14]. Secondly, the “information size” (i.e. number of trial participants needed for a reliable meta-analysis) should be at least as large as for an appropriately powered trial[9] to reach firm evidence. An “information size” estimate (based on plausible treatment effects) should be done as part of the protocol for a meta-analysis[8]. The use of TSA for calculation of an estimate RIS helps in the objective of deciding whether the benefits or risks of placing a mesh in the repair of a PVH or IH are derived from evidence firm enough to be accepted or rejected. After estimate of TSA, this meta-analysis shows firm evidence of the efficacy of mesh for preventing hernia recurrence. However, in relation to complications either for the general analysis, PVH and IH subgroups or high quality studies, a sufficient information size for all cases is lacking or an adequate calculation cannot be performed and, therefore, it is not possible to estimate whether the number of patients included is sufficient to evaluate potential mesh-related complications conclusively. Additional RCTs of high quality specifically analyzing separately the subgroups of IH, PVH, different types of meshes, or clean versus contaminated surgical fields are needed.

## Conclusion

The use of a nonabsorbable synthetic mesh in clean elective PVH or clean elective IH repair shows an advantage over simple suture to reduce the short- and mid-term risk of recurrent hernia. However, evidence regarding postoperative mesh-related complications remains inconclusive.

## Supporting information

S1 File PRISMA checklist. The checklist for reporting systematic reviews, as recommended by the PRISMA Statement.
(DOC)

S2 File Study protocol. (DOCX)

## References

[1] López-Cano M, Barreiro Morandeira F. Prosthetic material in incisional hernia surgery. Cir Esp. 2010;88(3):152–157. doi: 10.1016/j.ciresp.2009.12.015 20202628 10.1016/j.ciresp.2009.12.015

[2] Helgstrand F. National results after ventral hernia repair. Dan Med. 2016;63(7):B5258. 27399983

[3] Pereira JA, López-Cano M, Hernández-Granados P, Feliu X. Initial results of the National Registry of Incisional Hernia. Cir Esp. 2016;94(10):595–602. doi: 10.1016/j.ciresp.2016.09.008 27884387 10.1016/j.ciresp.2016.09.008

[4] Kokotovic D, Bisgaard T, Helgstrand F. Long-term recurrence and complications associated with elective incisional hernia repair. JAMA. 2016;316(15):1575–1582. doi: 10.1001/jama.2016.15217 27750295 10.1001/jama.2016.15217

[5] Nguyen MT, Berger RL, Hicks SC, Davila JA, Li LT, Kao LS, et al Comparison of outcomes of synthetic mesh vs suture repair of elective primary ventral herniorrhaphy: A systematic review and meta-analysis. JAMA Surg. 2014;149(5):415–421. doi: 10.1001/jamasurg.2013.5014 24554114 10.1001/jamasurg.2013.5014

[6] Mathes T, Walgenbach M, Siegel R. Suture versus mesh repair in primary and incisional ventral hernias: A systematic review and meta-analysis. World J Surg. 2016;40(4):826–835. doi: 10.1007/s00268-015-3311-2 26563217 10.1007/s00268-015-3311-2

[7] Evidence-based Medicine Working Group. Evidence-based medicine. A new approach to teaching the practice of medicine. JAMA. 1992;268(17):2420–2425. 1404801 10.1001/jama.1992.03490170092032

[8] Roberts I, Ker K, Edwards P, Beecher D, Manno D, Sydenham E. The knowledge system underpinning healthcare is not fit for purpose and must change. BMJ. 2015;350:h2463 doi: 10.1136/bmj.h2463 26041754 10.1136/bmj.h2463

[9] Brok J, Thorlund K, Gluud C, Wetterslev J. Trial sequential analysis reveals insufficient information size and potentially false positive results in many meta-analyses. J Clin Epidemiol. 2008;61(8):763–769. doi: 10.1016/j.jclinepi.2007.10.007 18411040 10.1016/j.jclinepi.2007.10.007

[10] Moher D, Liberati A, Tetzlaff J, Altman DG. Preferred reporting items for systematic reviews and meta-analyses: The PRISMA statement. Ann Intern Med. 2009;151(4): 264–269, W64 19622511 10.7326/0003-4819-151-4-200908180-00135

[11] Higgins JP, Altman DG, Gotzsche PC, Jüni P, Moher D, Oxman AD, et al The Cochrane Collaboration’s tool for assessing risk of bias in randomized trials. BMJ.2011;343:d5928 doi: 10.1136/bmj.d5928 22008217 10.1136/bmj.d5928 PMC3196245

[12] DerSimonian R, Laird N. Meta-analysis in clinical trials revisited. Contemp Clin Trials. 2015;45(PtA):139–145. 26343745 10.1016/j.cct.2015.09.002 PMC4639420

[13] Deeks JJ, Higgins JPT, Altman DG. Analysing data and undertaking meta-analyses In: Higgins JPT, Green S, eds. Cochrane Handbook for Systematic Reviews of Interventions. Chichester: John Wiley & Sons 2008:243–293.

[14] Higgins JPT, Thompson SG, Deeks JJ, Altman DG. Measuring inconsistency in meta-analyses. BMJ. 2003;327(7414):557–560. doi: 10.1136/bmj.327.7414.557 12958120 10.1136/bmj.327.7414.557 PMC192859

[15] Venclauskas L, Maleckas A, Kiudelis M. One-year follow up after incisional hernia treatment: Results of a prospective randomized study. Hernia. 2010;14(6):575–582. doi: 10.1007/s10029-010-0686-8 20567989 10.1007/s10029-010-0686-8

[16] Luijendijk RW, Hop WC, van den Tol MP, de Lange DC, Braaksma MM, IJzermans JN, et al A comparison of suture repair with mesh repair for incisional hernia. N Engl J Med. 2000;343(6):392–398. doi: 10.1056/NEJM200008103430603 10933738 10.1056/NEJM200008103430603

[17] Arroyo A, Garcia P, Pérez F, Andreu J, Candela F, Calpena R. Randomized clinical trial comparing suture and mesh repair of umbilical hernia in adults. Br J Surg. 2001;88(10):1321–1323. doi: 10.1046/j.0007-1323.2001.01893.x 11578284 10.1046/j.0007-1323.2001.01893.x

[18] Korenkov M, Sauerland S, Arndt M, Bograd L, Neugebauer EA, Troidl H. Randomized clinical trial of suture repair, polypropylene mesh or autodermal hernioplasty for incisional hernia. Br J Surg. 2002;89(1):50–56. doi: 10.1046/j.0007-1323.2001.01974.x 11851663 10.1046/j.0007-1323.2001.01974.x

[19] Burger JW, Luijendijk RW, Hop WC, Halm JA, Verdaasdonk EG, Jeekel J. Long-term follow-up of a randomized controlled trial of suture versus mesh repair of incisional hernia. Ann Surg. 2004;240(4):578–583. doi: 10.1097/01.sla.0000141193.08524.e7 15383785 10.1097/01.sla.0000141193.08524.e7 PMC1356459

[20] Polat C, Dervisoglu A, Senyurek G, Bilgin M, Erzurumlu K, Ozkan K. Umbilical hernia repair with the prolene hernia system. Am J Surg. 2005;190(1):61–64. doi: 10.1016/j.amjsurg.2004.09.021 15972174 10.1016/j.amjsurg.2004.09.021

[21] de Vries Reilingh TS, van Goor H, Charbon JA, Rosman C, Hesselink EJ, van der Wilt GJ, et al Repair of giant midline abdominal wall hernias: ‘‘components separation technique” versus prosthetic repair: Interim analysis of a randomized controlled trial. World J Surg. 2007;31(4):756–763. doi: 10.1007/s00268-006-0502-x 17372669 10.1007/s00268-006-0502-x PMC1913177

[22] Abdel-Baki NA, Bessa SS, Abdel-Razek AH. Comparison of prosthetic mesh repair and tissue repair in the emergency management of incarcerated para-umbilical hernia: a prospective randomized study. Hernia. 2007;11(2):163–167. doi: 10.1007/s10029-007-0189-4 17273815 10.1007/s10029-007-0189-4

[23] Ammar SA. Management of complicated umbilical hernias in cirrhotic patients using permanent mesh: Randomized clinical trial. Hernia. 2010;14(1):35–38. doi: 10.1007/s10029-009-0556-4 19727551 10.1007/s10029-009-0556-4

[24] Weber G, Baracs J, Horvath OP. ‘‘Onlay” mesh provides significantly better results than ‘‘sublay” reconstruction. Prospective randomized multicenter study of abdominal wall reconstruction with sutures only, or with surgical mesh–results of a five-years follow-up. Magy Seb. 2010;63(5):302–311. doi: 10.1556/MaSeb.63.2010.5.3 20965863 10.1556/MaSeb.63.2010.5.3

[25] Lal K, Laghari ZH, Laghari AA, Soomro E. A comparative study of anatomical repair versus mesh repair in paraumbilical hernia. Medical Channel. 2012;19(2):110–113.

[26] Schünemann H, Brożek J, Guyatt G, Oxman A, eds. Handbook for grading the quality of evidence and the strength of recommendations using the GRADE approach. [Updated October 2013].http://gdt.guidelinedevelopment.org/central_prod/_design/client/handbook/handbook.html#h.svwngs6pm0f2. Accessed date Abril 15, 2016.

[27] McCulloch P, Altman DG, Campbell WB, Flum DR, Glasziou P, Marshall JC, et al No surgical innovation without evaluation: The IDEAL recommendations. Lancet. 2009;374(9695):1105–1112. doi: 10.1016/S0140-6736(09)61116-8 19782876 10.1016/S0140-6736(09)61116-8

[28] Sedrakyan A, Campbell B, Merino JG, Kuntz R, Hirst A, McCulloch P. IDEAL-D: A rational framework for evaluating and regulating the use of medical devices. BMJ. 2016;353:i2372 doi: 10.1136/bmj.i2372 27283585 10.1136/bmj.i2372

[29] Pogue J, Yusuf S. Overcoming the limitations of current meta-analysis of randomised controlled trials. Lancet. 1998;351(9095):47–52. doi: 10.1016/S0140-6736(97)08461-4 9433436 10.1016/S0140-6736(97)08461-4

[30] Pogue JM, Yusuf S. Cumulating evidence from randomized trials: Utilizing sequential monitoring boundaries for cumulative meta-analysis. Control Clin Trials. 1997;18:580–593; discussion 661–666. 9408720 10.1016/s0197-2456(97)00051-2

[31] Flum DR, Horvath K, Koepsel T. Have outcomes of incisional hernia repair improved with time? A population-based analysis. Ann Surg. 2003;237(1):129–135. doi: 10.1097/01.SLA.0000041042.86225.9C 12496540 10.1097/00000658-200301000-00018 PMC1513979

[32] den Hartog D, Dur AH, Tuinebreijer WE, Kreis RW. Open surgical procedures for incisional hernias. Cochrane Database Syst Rev. 2008 7 16;(3):CD006438 doi: 10.1002/14651858.CD006438.pub2 18646155 10.1002/14651858.CD006438.pub2 PMC8924951

[33] Klinge U, Klosterhalfen B, Muller M, Schumpelick V. Foreign body reaction to meshes used for the repair of abdominal wall hernias. Eur J Surg. 1999;165(7):665–673. doi: 10.1080/11024159950189726 10452261 10.1080/11024159950189726

[34] Westphalen AP, Araújo AC, Zacharias P, Rodrigues ES, Fracaro GB, Lopes Filho Gde J. Repair of large incisional hernias. To drain or not to drain. Randomized clinical trial. Acta Cir Bras. 2015;30(12):844–851. doi: 10.1590/S0102-865020150120000009 26735057 10.1590/S0102-865020150120000009

