# Supplementary material for: Balancing mesh-related complications and benefits in primary ventral and incisional hernia surgery. A meta-analysis and trial sequential analysis
Source: PLoS One. 2018 Jun 6;13(6):e0197813. doi: 10.1371/journal.pone.0197813 (PMC5991361; doi:10.1371/journal.pone.0197813)
Supplement: S2 File — (DOCX) [file pone.0197813.s002.docx]

**STUDY PROTOCOL**

**Review title**

Balancing mesh-related complications and benefits in primary ventral hernia (PVH) and ventral incisional hernia (VIH) surgery. A Meta-analysis and Trial Sequential Analysis

**Review team**

Manuel López Cano, Abdominal Wall Surgery Unit, Department of General Surgery, Hospital Universtario de la Vall d'Hebron, Universidad Autonoma de Barcelona

Lidia A. Martin-Dominguez, Abdominal Wall Surgery Unit, Department of General Surgery, Hospital Universtario de la Vall d'Hebron, Universidad Autonoma de Barcelona.

José Antonio Pereira, Department of Surgery, Parc de Salut Mar, Experimental and Health Science, Universitat Pompeu Fabra, Barcelona

Manuel Armengol-Carrasco, Abdominal Wall Surgery Unit, Department of General Surgery, Hospital Universtario de la Vall d'Hebron, Universidad Autonoma de Barcelona

Josep García-Alamino, Department of Primary Health Care Sciences, University of Oxford, Oxford, United Kingdom.

**Funding sources/sponsors**

Department of General Surgery, Hospital Universitari Vall d’Hebron, Barcelona

**Conflicts of interest**

None

**Review methods**

*Review objectives*

To compare placement of a mesh versus simple suture for recurrence and postoperative complications in PVH and VIH repair.

*Searches*

The following databases will be searched: MEDLINE (PubMed), SCOPUS, CINAHL, WOK (Web of Knowledge), and Google Scholar. Inclusion criteria for the search: The search will be limited to randomized controlled trials (RCTs) with a maximal sensitive strategy. No language or publication date restrictions will be applied. Search

Search strategy will be based on combinations of Medical Subject Heading (MeSH) terms and text words for each database.

*Search strategy (for Medline)*

"Search ((((((((""Hernia, Incisional"" OR ""Hernias, Incisional"" OR ""Incisional Hernias"" OR ""Postoperative Hernia"" OR ""Hernia, Postoperative"" OR ""Hernias, Postoperative"" OR ""Postoperative Hernias"") AND ( ( Meta-Analysis[ptyp] OR Randomized Controlled Trial[ptyp] OR Review[ptyp] OR systematic[sb] ) AND ( English[lang] OR Spanish[lang] OR French[lang] OR German[lang] ) ))) OR ((""Hernias, Ventral"" OR ""Ventral Hernias"" OR ""Ventral Hernia"") AND ( ( Meta-Analysis[ptyp] OR Randomized Controlled Trial[ptyp] OR Review[ptyp] OR systematic[sb] ) AND ( English[lang] OR Spanish[lang] OR French[lang] OR German[lang] ) ))) AND ( ( Meta-Analysis[ptyp] OR Randomized Controlled Trial[ptyp] OR Review[ptyp] OR systematic[sb] ) AND ( English[lang] OR Spanish[lang] OR French[lang] OR German[lang] ) ))) AND ((((""mesh"") OR ""surgical meshes"") OR ""surgical mesh"") AND ( ( Meta-Analysis[ptyp] OR Randomized Controlled Trial[ptyp] OR Review[ptyp] OR systematic[sb] ) AND ( English[lang] OR Spanish[lang] OR French[lang] OR German[lang] ) ))) AND ( ( Meta-Analysis[ptyp] OR Randomized Controlled Trial[ptyp] OR Review[ptyp] OR systematic[sb] ) AND ( English[lang] OR Spanish[lang] OR French[lang] OR German[lang] ) ))"

*Condition or domain being studied*

The domain being studied is to assess the effectiveness and postoperative complications of placement of a nonabsorbable synthetic mesh versus simple suture for treating patients with PVH or VIH undergoing surgical repair.

*Participants/population*

The patient population being studied: patients who have undergone PVH or VIH operation, The repair will be done with nonabsorbable synthetic mesh, adult patients (>18 years old), patients without intolerance to synthetic meshes, life expectancy more than 12 months.

*Intervention, exposure*

The intervention being reviewed is the placement of a nonabsorbable synthetic mesh in adult patients undergoing PVH or VIH surgery.

*Comparator/control*

Patients with PVH or VIH hernia repair receiving a nonabsorbable synthetic mesh can be compared with patients with PVH or VIH repair without receiving a nonabsorbable synthetic mesh (simple suture).

*Types of study to be included initially*

Randomized Controlled Trials (RCTs)

*Context*

We established the inclusion criteria for study selection according to the PICOS approach. The population consisted of patients with a PVH or VIH undergoing hernia repair. The intervention consisted of placement of a nonabsorbable synthetic mesh, regardless of mesh location, surgical technique, hernia characteristics or surgical setting for the repair of a PVH or VIH of the abdominal wall, compared to primary suture of the PVH or VIH without mesh.

*Primary outcome*

Primary outcome will be the incidence of recurrence of PVH or VIH diagnosed on clinical grounds by physical examination or radiological evaluation (computed tomography [CT] scan or ultrasonography).

*Secondary outcome*

Secondary outcomes will be wound infection, hematoma, seroma, postsurgical pain, length of operation for each type of surgical procedure, and health-related quality of life.

*Data extraction*

Selecting studies will be done based on inclusion and exclusion criteria. Only RCTs will be included. Extracting data will be (independently) done by at least two researchers. Data will be extracted with Rev Manager 5.3 software . An initial (general) meta-analysis pooling the results from PVH and VIH for the primary outcome it´s planned. Additionally, pooled postoperative complications will be meta-analyzed in separate and also grouped to estimate the overall effect of complication events.

Sensitive analysis within subgroups (i.e. PVH and VIH) for recurrence and postoperative complications (separate and grouped) it´s planned. Meta-analysis that combine other subgroups (mesh location, hernia characteristics or surgical setting) are also planned. Sequential multiplicity (repeated updates) and sparse data increased the risk of type I error and to control this a Trial Sequential Analysis (TSA) will be performed. The number of researchers involved is five.

*Risk of bias (quality) assessment*

The randomization method and blind assessment of results will be used to assess the risk of bias (quality) of studies included in the review.

*Strategy for data synthesis*

A summary of findings table will be made including all studies and their qualitative assessment. For the meta-analysis (quantitative assessment) only randomized studies will be included. Data will be extracted from the published manuscripts.

**General information**

*Type of review*

Meta-analysis and Trial Sequential analysis

*Dissemination plans*

The results will be published in a journal of the specialty (if possible)
